# Forty years in the field: reproductive biotechnologies shaping genetic progress in cattle in France

**DOI:** 10.1590/1984-3143-AR2026-0058

**Published:** 2026-08-28

**Authors:** Serge Lacaze

**Affiliations:** 1 Auriva Biotechnologies, Auriva-Élevage, France

**Keywords:** embryo transfer, OPU-IVP, cattle breeding, genomic selection, embryo biopsy, reproductive biotechnology, genetic improvement

## Abstract

Over the past four decades, reproductive biotechnologies have profoundly transformed cattle breeding by accelerating genetic progress and enabling the dissemination of elite genetics. In this article, I present a perspective based on more than 40 years of practical experience in embryo technologies within Auriva-Elevage, a cooperative organization serving 30,000 farmers in southern France. The development of embryo transfer in France was closely linked to genetic and sanitary challenges, particularly the introduction of North American Holstein genetics and the restrictions on live animal imports due to infectious diseases such as Infectious Bovine Rhinotracheitis. These constraints stimulated the development of national expertise in embryo transfer. Over the years, our team has implemented and adapted a wide range of reproductive technologies including *in vivo* embryo production and embryo transfer, cryopreservation, embryo sexing, ovum pick-up (OPU), *in vitro* embryo production (IVP), embryo biopsy, genomic evaluation of embryos, and laser-assisted biopsy techniques. The genomic revolution dramatically increased the strategic value of OPU-IVP for the rapid multiplication of elite donor females. In addition to technological developments, the success of these programs has depended heavily on internal training, collaboration with national organizations such as ELIANCE (previously UNCEIA, ALLICE) and research institutes including INRAE and Toulouse veterinary school, as well as strong international exchanges through scientific networks. Practical examples such as the use of embryo biopsy to prevent genetic diseases demonstrate the applied value of these technologies in breeding programs. This review highlights the technical evolution, organizational structures, and human expertise that have shaped the implementation of reproductive biotechnologies in cattle breeding and discusses the importance of anticipating future needs to ensure continued genetic progress.

## Introduction

What a surprise and what an honor it was to receive last November 2025 the mail of Dr. Hilde AARDEMA, our current AETE President, with such kind words : “*Dear Dr. Serge Lacaze, dear Serge, It is with great pleasure to announce that we selected you as our next Pioneer Award winner for the AETE 2026. We very much hope that you will accept this invitation*”.

It reminded me the list of distinguished previous Pioneer Award recipients, but also I realized how proud I was to represent and dedicate this prestigious Pioneer Award to all practitioners and managers of embryo production teams. The connection between all stakeholders: researchers, academics, students, and practitioners is the key to the success of any technique. Thank you for this honor.

Reproductive biotechnologies have become essential tools for accelerating genetic progress in modern cattle breeding while taking in to account health and longevity traits that contribute to more sustainable breeding. Among these technologies, embryo transfer and *in vitro* embryo production have played a central role by allowing the rapid multiplication of elite genetic lines, facilitating international genetic exchanges and preventing genetic diseases through genomic-based markers (Ponsart et al., 2014).

**Figure 1:**
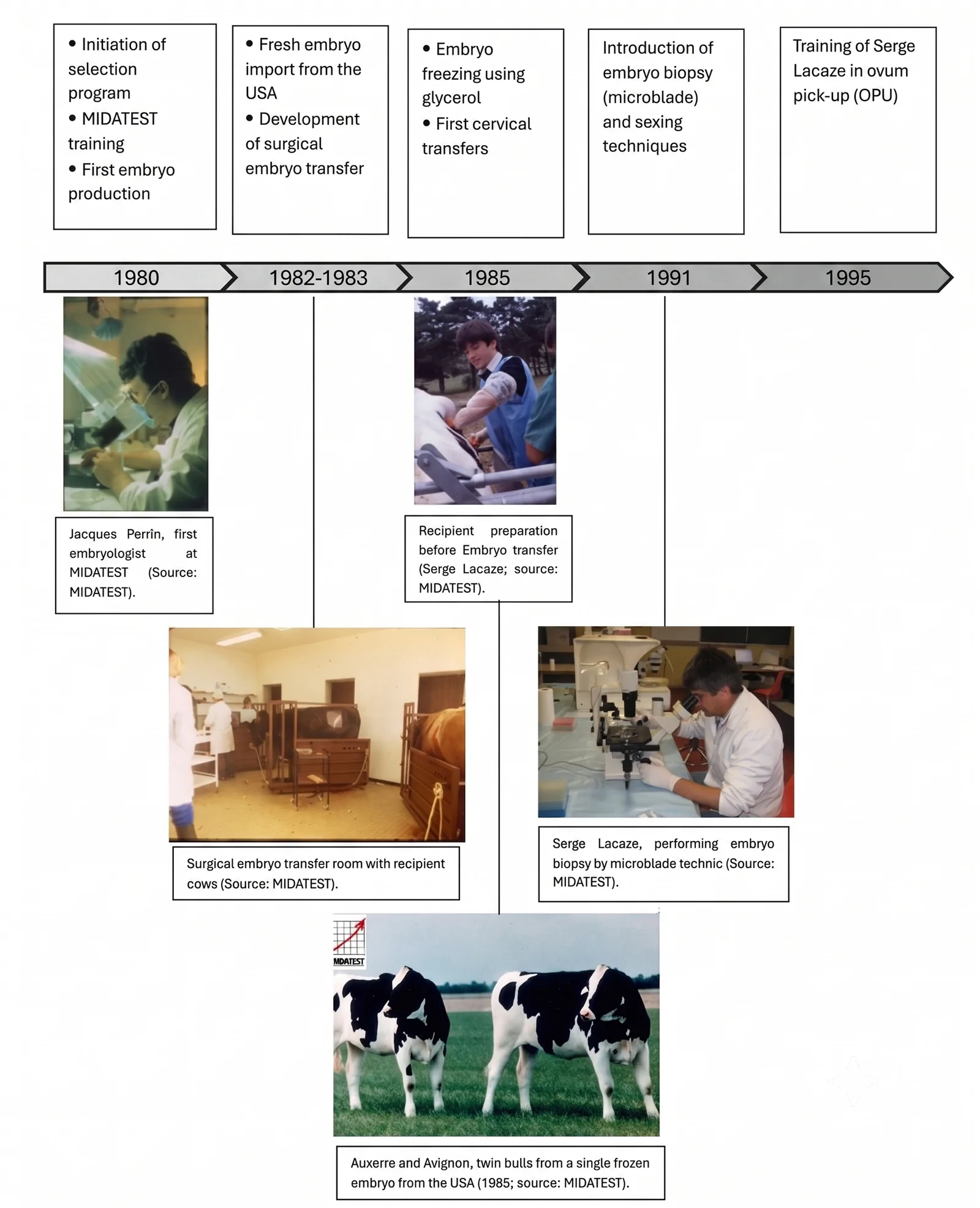
Timeline illustrating the main biotechnological advances in cattle reproduction in France from 1980 to 1995.

**Figure 2:**
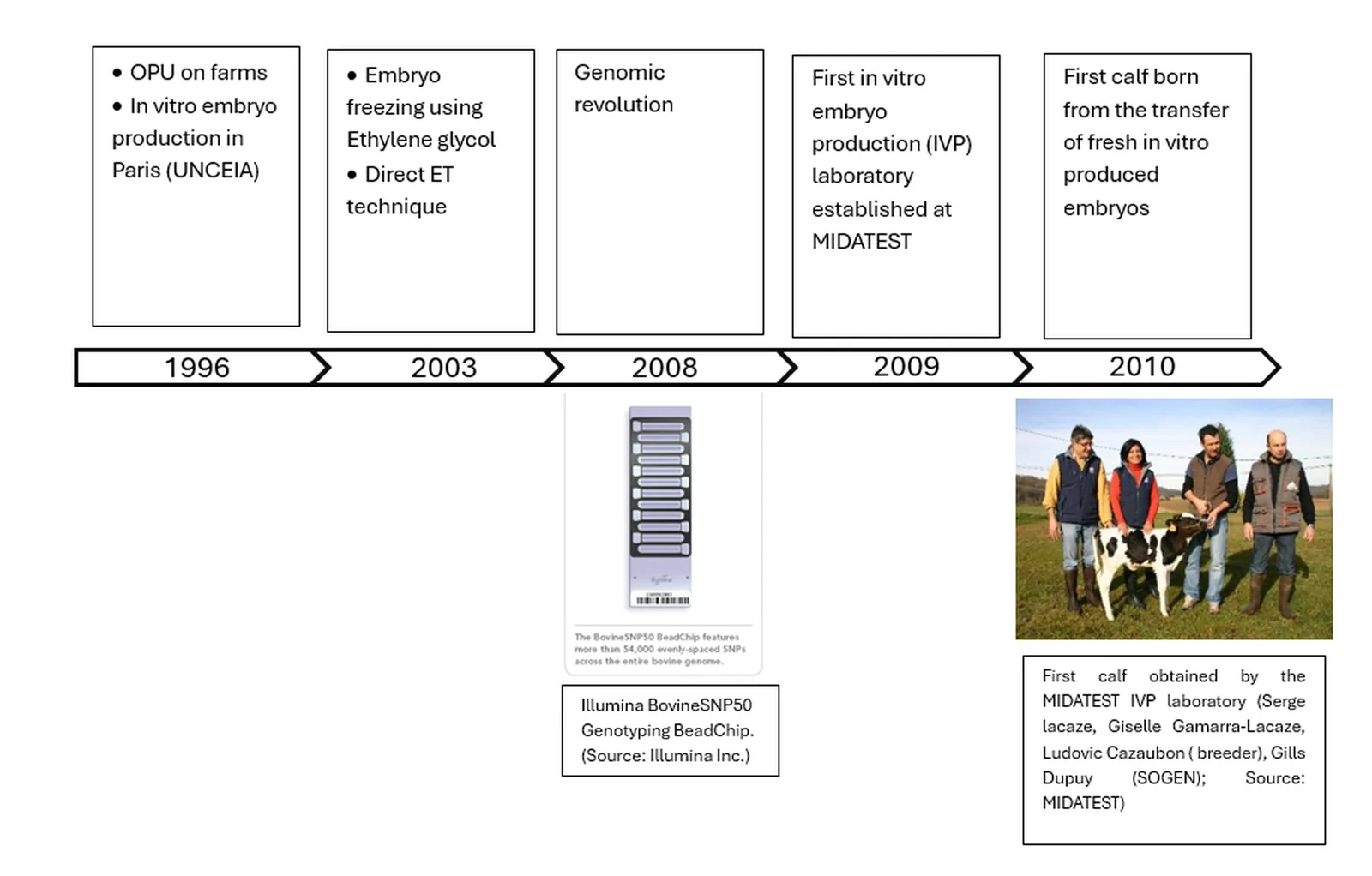
Timeline illustrating the main biotechnological advances in cattle reproduction in France from 1996 to 2010.

**Figure 3:**
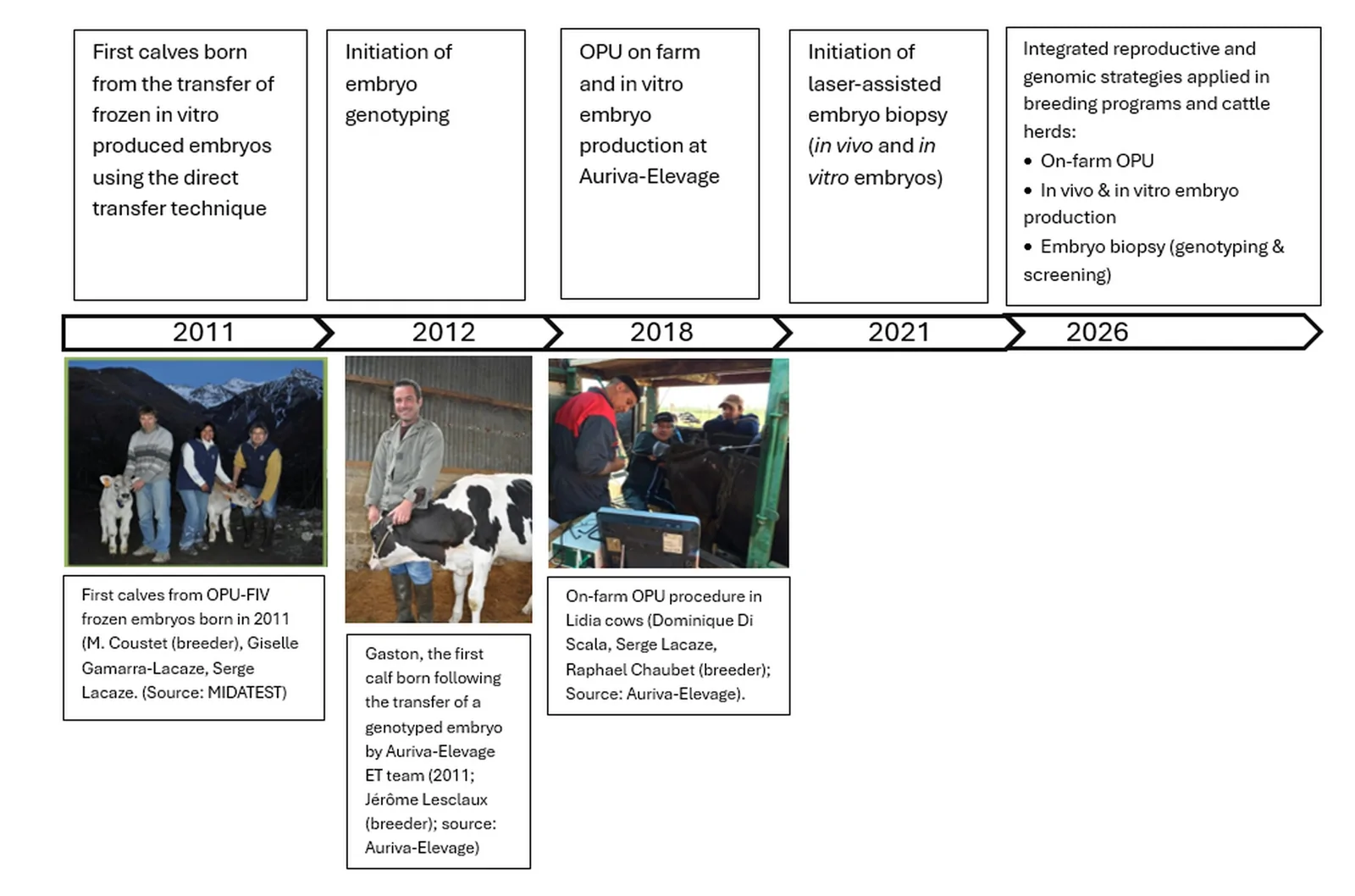
Timeline illustrating the main biotechnological advances in cattle reproduction in France from 2011 to 2026.

When I began working in embryo transfer in 1982, this field was still in its early stages in France. At that time, the techniques were largely experimental, equipment was limited, and protocols were still being defined (Thibier, 1990; Lacaze et al., 1992). Over the past forty years, I have had the opportunity to witness the gradual transformation of these technologies from experimental procedures into routine tools applied by breeding organizations and farmers (Moore and Hasler, 2017). A detailed timeline of the main biotechnological developments over the past forty years in France is presented in Figures 1, 2 and 3.

Today, our team at Auriva-Elevage produces approximately 8,000 embryos annually, both *in vivo* and *in vitro*, including around 800 embryos biopsied for genomic analysis (Auriva-Elevage, rapport d’activité 2024-2025). This level of production reflects decades of technical refinement, organizational development, and collaboration between practitioners, researchers, and breeding organizations.

Auriva-Elevage (MIDATEST until 2016) represents a union of artificial insemination cooperatives responsible for managing fourteen cattle breeding programs and two goat breeding programs for 30,000 farmers in southern France. Within this cooperative structure, reproductive biotechnologies have progressively been integrated into breeding strategies to enhance genetic gain and improve herd productivity.

In this article, I provide a retrospective perspective on the development of embryo technologies in France and within the Auriva-Elevage team. My objective is not only to describe the technological milestones that have shaped this field, but also to highlight the organizational and human factors that have been essential for their successful implementation.

## The origin of embryo transfer in France

The development of embryo transfer in France occurred during a period of major transformation in dairy cattle breeding. While embryo transfer techniques were initiated in North America in 1975, technically enabled by the introduction of prostaglandins for controlling the estrous cycle in cows (Hasler, 2014), French breeding companies made an important genetic decision by focusing on the North American-type Holstein cow specialized for milk. This led to the absorption of the FFPN (Française Frisonne Pie Noire) breed, a dual-purpose milk-and-meat breed, which no longer corresponded to the production objectives of many Auriva member breeders.

To introduce these new lines, two approaches were considered: the use of imported semen as the primary method, and the introduction of high-genetic-value females to also produce males to strengthen the breeding programs for these breeds. The promising development of embryo transfer in North America provided the opportunity to multiply these animals efficiently. However, the implementation of genetic improvement has always been closely linked to sanitary conditions. In 1981, the closure of borders to the importation of live animals due to IBR (Infectious Bovine Rhinotracheitis) prevented the use of this approach (European Union, 1964; Badin de Montjoye, 2003). During this period, France maintained restrictive policies regarding bovine imports until the liberalization of intra-Community trade in 1993. Under these circumstances, the importation of embryos became the most feasible strategy for introducing foreign genetics while maintaining strict sanitary control, with strict control over both the recipient females and the resulting offspring to ensure that IBR was not transmitted via embryos. (Thibier and Nibart, 1987; Singh, 1988; Stringfellow and Seidel, 1990; Thibier, 1988, 1990).

For approximately ten years, all imported embryos were transferred into specialized recipient stations where both recipients and resulting offspring were monitored to verify that embryos did not transmit the disease. This national program was coordinated by Bernard Guérin from the Laboratoire National de Contrôle des Reproducteurs and Michel Thibier **(**Thibier and Guérin,1993). This experience confirmed that embryos produced in compliance with the International Embryo Technology Society (IETS) production standards constitute a reliable method for ensuring animal health in international genetic exchanges. The absence of disease transmission confirmed that embryos produced according to sanitary standards defined by the IETS represented a safe method for international genetic exchange (IETS,1987).

In 1979, Jacques Perrin of MIDATEST (Figure 1) visited several North American embryo transfer teams to observe their techniques and bring back practical knowledge that could be implemented in southern France. During this period, we also had the honor of welcoming and exchanging experiences with Jacques Testart, a pioneer in human in vitro fertilization. In his 1986 book, *L’Œuf transparent*, he referred to the discussions we had together (Testart, 1986).

By 1980, four French cooperative teams (URCEO, OGER, France Embryon and MIDATEST) were assembled under the coordination of UNCEIA, led by Michel Thibier and Michel Nibart, who played a central role in this national initiative. At that time, the main objective was to master embryo transfer and implement it under field conditions within breeding programs (Nibart and Bouyssou, 1981; Thibier and Nibart, 1992).

These teams collaborated and exchanged knowledge to develop proficiency in this emerging and transformative technology, which was critical for the advancement of breeding programs in France and for the dissemination of superior female genetics (Lacaze et al., 1992). Their work focused on:

- Definition of superovulation protocols
- Selection of equipment for embryo collection and transfer
- Determination of the collection medium
- Establishing methods for filtering the collection medium.

Following this national program, the four teams further developed and implemented the technique within their respective geographic regions.

In the same year, a specific embryo classification system based on morphological defects was implemented at MIDATEST, prior to the adoption of the IETS criteria. This classification was later found to correspond to IETS quality grade 1. Embryos presenting no defects (“0 defects”, excellent) or a single defect (“1 defect”, good) were classified as IETS quality 1, whereas embryos presenting two defects (regular) were classified as quality 2 (IETS, 1987). At the moment MIDATEST classification was essential for improving the evaluation of embryos after thawing (glycerol cryopreservation followed by stepwise dilution of glycerol during thawing). Having an exact description of the embryo at the time of freezing (stage and defects) allowed us to accurately assess its post-thaw development and determine whether the cryopreservation process had induced any damage.

Each embryo was carefully evaluated and systematically documented through detailed drawings, establishing a form of “embryography” that enabled the standardization of embryo assessment among practitioners (Bourdin et al., 2008).

## A highly efficient national research organization adapted to Industry needs

Following adoption of the “French Livestock Law” (France, 1966; Cornu, 2024), artificial insemination cooperatives in France organized themselves at the national level to pool financial, technical, and human resources. This national cooperative structure was initially created as UNCEIA (Union Nationale des Coopératives Agricoles d’Elevage et d’Insémination Animale), later renamed Allice, and today known as Eliance (Eliance, 2022). The objective of this organization was to support the development of livestock genetics and reproductive technologies through coordinated actions among artificial insemination cooperatives across the country (Allice, 2017).

This national institute ensured the representation of French breeding cooperatives at both national and international levels with close ties with the Ministry of Agriculture, veterinary authorities, and international organizations. This institute aimed to shape sanitary regulations, facilitate the exchange of genetic material, and establish reference populations for genomic selection and also provided legal, technical, and social support to its members. By enhancing coordination across the livestock sector and maintaining strong collaborations with research institutes, particularly INRAE, it enabled the development of national and international research programs in genetics and reproductive biotechnologies (Conseil de la concurrence, 2004).

An important component of this organization has been the development of applied research programs responding directly to the needs expressed by cooperative members. Several technological innovations in reproductive biotechnology emerged from these collaborative initiatives. For example, the development of embryo genotyping programs was conducted under the scientific responsibility of Patrice Humblot, Claire Ponsart and Daniel Lebourhis (Ponsart et al., 2014), while advances in *in vitro* fertilization were achieved through collaborative work involving Brigitte Leguienne and Catherine Joly (Le Guienne and Thibier, 1988; Guyader-Joly et al.,2000).

This collective organizational framework has provided a level of efficiency that would have been difficult to achieve individually by separate cooperatives. By pooling resources, expertise, and research efforts, the French system has enabled the rapid development and implementation of advanced reproductive technologies. Embryo technologies have particularly benefited from this coordinated national structure, which has facilitated both technological innovation and the dissemination of these methods to breeders.

Michel Thibier, a visionary, recognized from the very beginning the need to promote European-level exchanges in embryo technology. In 1984, he founded the AETE, which went on to achieve the well-known success it enjoys today. During the first fifteen years, the association was composed mainly of practitioners working in embryo transfer. Subsequently, reproduction specialists, researchers, and students joined the association, contributing new scientific knowledge and strengthening its scientific dimension (Thibier, 2014).

Since then, I have had the privilege of actively participating in the AETE alongside many distinguished colleagues, attending 35 of the 41 annual meetings, organizing workshops, and serving on the board for 10 years (2006–2015), contributing to the strategic direction and international influence of the association. My main objective on the board was to ensure a good balance between scientific presentations and practical workshops of primary interest to practitioners. Following this period, from 2015 onward, I have also been serving as the collector of French embryo transfer activities for the AETE, contributing to the documentation and monitoring of national practices and developments in the field. Some of the workshops that I had the opportunity to moderate and/or present are listed in Table 1.

**Table 1:** Selected Workshops Attended by the Author at AETE Congresses as coordinator or speaker (1995–2024)

**Year**	**AETE Meeting**	**Country**	**Topic / Workshop Focus (coordinator, C; speaker, S)**
1995	Lyon	France	Embryo sexing (C; S)
2006	Zug	Switzerland	Materials and methods used for embryo collection (C;S)
2008	Pau	France	Assisted reproduction and genetic selection in beef breeds (C; S)
2009	Poznan	Poland	Donor cow management (S)
2012	Saint-Malo	France	Oocyte collection: what is new? (S)
2014	Dresden	Germany	Genotyping of embryos (C; S)
2016	Barcelona	Spain	Sonography, Doppler, new tools for donor and recipient selection (C)
2017	Bath	United Kingdom	Selection and pre-treatment of donor animals before oocyte/embryo collection (flushing and OPU) (S)
2017	Bath	United Kingdom	Micromanipulation (C; S)
2023	Heraklion	Greece	Practitioners’ forum: SOPs for donors and recipients (S)
2024	Brescia	Italy	Embryo therapy to increase the conception rate (S)

This extensive involvement has allowed me to contribute to and benefit from the latest advances in practical reproductive biotechnology, fostering knowledge exchange and professional development across Europe.

## Technical evolution within the Auriva Biotech Team

The technical evolution of reproductive biotechnologies within our team occurred progressively over several decades.

In the early 1980s, our work focused on mastering basic procedures of embryo transfer, including donor and recipient preparation, superovulation treatments, embryo collection using double-lumen catheters, embryo recovery by sedimentation after embryo collection, flushing medium preparation, and embryo classification based on numbers of defects. At that time, many embryo transfers were performed surgically in recipient stations, although we progressively developed non-surgical cervical transfer techniques using equipment adapted from artificial insemination devices (Nibart and Bouyssou, 1981; Mapletoft and Hasler, 2005).

Between 1982 and 1983, we performed the first transfers of fresh embryos imported from Wisconsin, USA. These transfers were carried out surgically and resulted in pregnancy rates exceeding 55%, which was highly encouraging at that time (Figure 1). Similar conception rates were also reported in surgical embryo transfer work conducted in the United States (Schneider et al., 1980).

**Figure 4:**
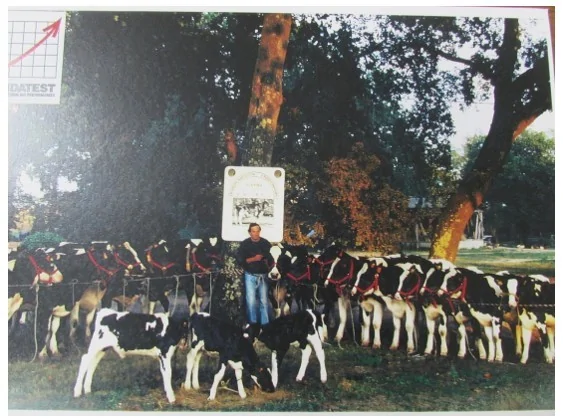
Ocarina cow (EARL de Banos) with her offspring following MOET (multiple ovulation and embryo transfer). Source: MIDATEST (2010).

In 1983, we introduced mobile laboratories, which enabled embryo collections to be performed directly on farms across southern France. This innovation significantly increased the accessibility of embryo transfer for breeders and facilitated the practical application of advanced reproductive technologies under field conditions. An example of the work we performed is the multiplication of an elite cow, Ocarina, via embryo transfer, as illustrated in Figure 4.

By 1985, frozen embryos preserved using glycerol-based cryopreservation techniques were introduced through importation from the USA. These embryos, frozen in glass ampoules, required a six-step rehydration procedure under a stereomicroscope prior to transfer (Massip et al., 1979), highlighting the technical constraints associated with early cryopreservation methods (Bielanski et al., 1986). Offspring produced from this work are illustrated in Figure 1.

In the same year, substantial progress was achieved at the Auriva level in the standardization of glycerol-based embryo cryopreservation in 0.25 mL French straws (Massip et al., 1987). However, an internal audit of cryopreservation practices identified that prolonged intervals between embryo collection and freezing adversely affected the consistency and reproducibility of results. Optimization of laboratory organization and workflow, particularly through the reduction of collection-to-freezing time, enabled the successful standardization of the glycerol freezing protocol, resulting in improved reliability and reproducibility of outcomes.

By 1990, improvements in embryo transfer equipment, including the development of standardized transfer systems such as the IMV side-delivery sheath (Curtis, 2015) and the introduction of embryo filtration devices for recovery procedures, greatly facilitated embryo identification, handling, and transfer. These technological advances contributed to the simplification and widespread adoption of non-surgical embryo transfer techniques, which had previously been developed in the USA (Bowen et al., 1978).

**Figure 5:**
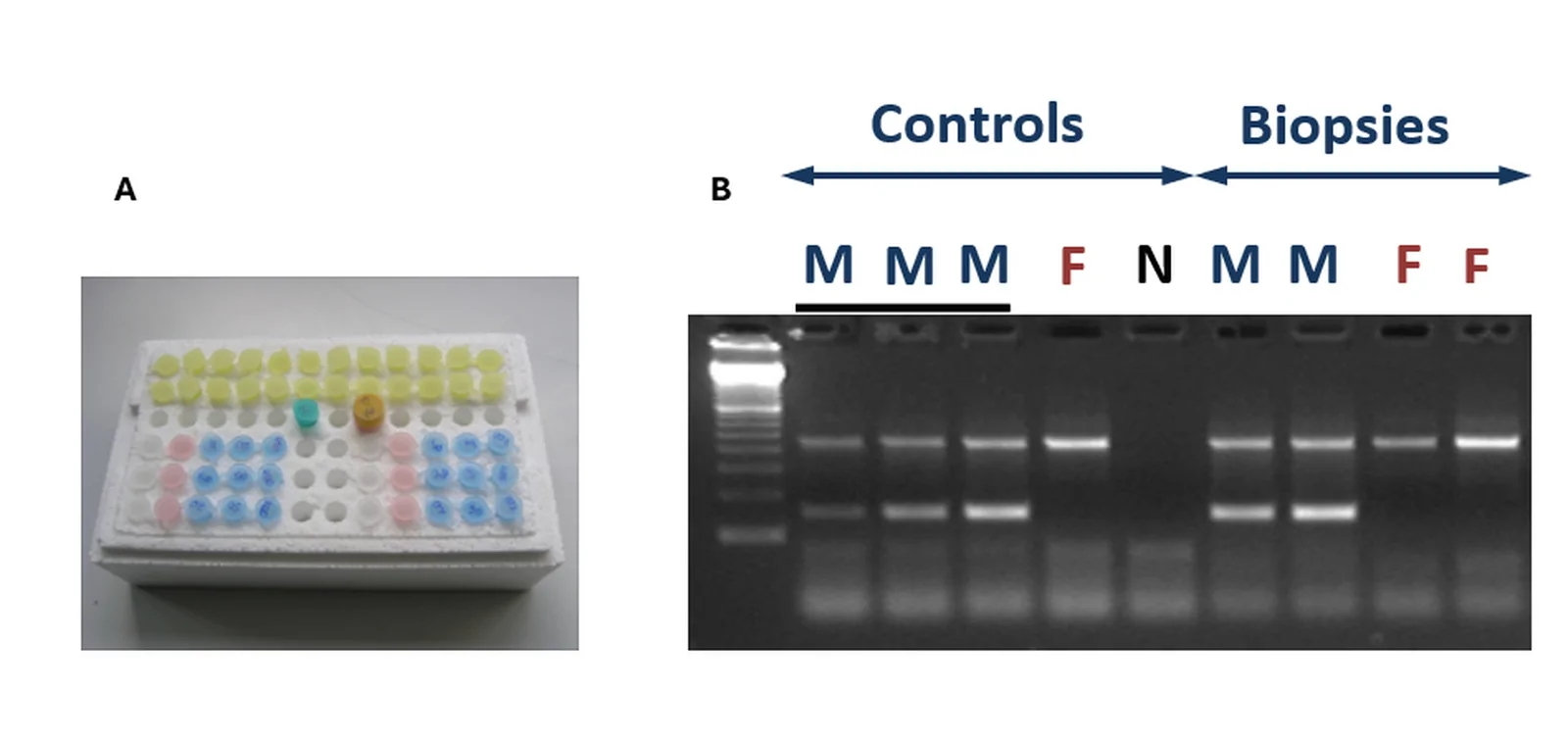
Embryo sexing tools and representative results. (A) UNCEIA Sexing Kit; (B) Electrophoresis results of embryo sexing samples with a negative control. F: Female; N: Negative control (no sample); M: Male. Source: MIDATEST (2010).

In 1991, breeders began requesting female embryos from valuable donors that were not part of official breeding schemes. At that time, sexed semen did not exist. In collaboration with the technical services of INRA and UNCEIA, we developed an embryo sexing technique based on the identification of a specific sequence of the Y chromosome (INRA license 1987) and the use of PCR technique after obtaining licensing agreements with Perkin-Elmer (Figure 5). This method allowed embryos to be analyzed and transferred fresh approximately six to seven hours after collection (Lacaze et al., 2008).

By 1993, the initiation of *in vitro* fertilization (IVF) started in France, with the implementation of two IVF laboratories launched by our national union in Paris and in Châteauvillain (Rhône-Alpes region). This milestone marked the beginning of a new era for reproductive biotechnology in the country, combining national coordination with cutting-edge laboratory techniques (Le Guienne and Thibier, 1988; Marquant-Le Guienne and Humblot, 1998; Guyader-Joly et al., 2000).

In 1995, I was trained OPU technology at Utrecht University by Dr. Pieterse, following the methodology he developed (Pieterse et al., 1988, 1991). Following this training, we acquired our own OPU equipment and established the first version of our i*n vitro* fertilization laboratory in Denguin in the south of France. This marked a major step for our team, allowing us to implement OPU and *in vitro* maturation under our direct supervision and to begin developing practical protocols for both station and on-farm applications.

Between 1998 and 2003, we gradually developed OPU procedures both in stations and on farms, while keeping oocyte maturation in our laboratory. At the end of maturation, oocytes were transported by airplane to the laboratory of our National Union (UNCEIA) in Paris, under the supervision of Brigitte Leguienne, and embryos were returned by airplane seven days later for fresh transfer, averaging one embryo per OPU session (Lacaze et al., 1997). During this period, we used the technique OPU-IVP moderately for two main reasons: embryo production rates were still low, and the accuracy of the genetic value of donor cows was insufficient to allow large-scale multiplication of selected genetics. Since genomic selection was not yet available, it was preferable to moderately multiply several cows of similar genetic level, with a reliability of approximately 0.25–0.30 of CD (coefficient of determination), to expand the selection base. Additionally, the selection of heifers was based only on pedigree index, which had limited precision.

In 2002, we evaluated a direct transfer (DT) approach based on the glycerol–sucrose protocol developed by Massip (Massip and Van der Zwalmen, 1984). Although this method proved effective, its practical application in the field was constrained by the need for successive rehydration steps prior to transfer. In 2003, we subsequently introduced direct embryo transfer using ethylene glycol (EG), which significantly simplified the procedure and enabled transfers to be performed by insemination technicians without the need for embryo visualization (Dochi et al., 1998; Voelkel and Hu, 1992; Nibart and Humblot, 1997). Compared with glycerol-based protocols, EG offered greater practicality under field conditions, improving both efficiency and consistency in embryo handling and transfer (Massip, 2001).

From 2005 to 2006, we focused on producing oocytes via OPU for research programs in collaboration with the National Veterinary School of Toulouse (ENVT), INRA, and UNCEIA. The studies evaluated the effects of repeated OPU on ovarian stimulation response and the proportion of diploid oocytes after *in vitro* maturation in Blonde d’Aquitaine cows (Bonnet-Garnier et al., 2008a, 2008b). These programs allowed us to maintain mastery of OPU techniques while the field of genomics was rapidly developing.

## The genomic revolution and the development of *in vitro* embryo production (IVP) at Auriva-Elevage

In 2008, the advent of genomics (Project “SAM2”; 2^nd^ generation of markers assisted selection) revolutionized our work. The accuracy of genetic evaluations for both male and female cattle increased to levels comparable to those obtained through progeny testing. Consequently, the potential for large-scale multiplication of offspring from elite donor cows renewed interest in OPU-IVP technologies.

In 2009, an OPU-IVP laboratory was established in Denguin upon the arrival of Giselle Gamarra-Lacaze and the support of Brigitte Le Guienne (UNCEIA). This OPU-IVP laboratory was the first cattle IVP laboratory developed by a breeding company in France to independently perform embryo production (MIDATEST press release, 2010). The technique rapidly yielded positive results and was quickly integrated into selection programs. The development of IVF protocols using sexed semen, achieving 48% Grade 1 blastocyst rates compared with 50% using conventional semen, was particularly valued by breeders, as it provided the opportunity to produce female embryos from their best cows (Gamarra et al., 2010, 2011, 2012, 2015a, 2016). Figure 2 shows the first calf born in 2010 at the MIDATEST IVP laboratory from a fresh embryo produced using sexed semen.

We considered that the success of *in vitro* embryo production had to be accompanied by efficient cryopreservation of *in vitro*–produced embryos and satisfactory pregnancy rates, as the main limitation was the availability of recipient females. By 2010, we had developed a direct transfer technique for the successful cryopreservation of *in vitro* embryos, enabling the distribution of frozen embryos with consistent pregnancy outcomes (45%), with the first calves born in 2011, as illustrated in Figure 3 (Lacaze et al., 2012).

This development enabled the hosting of high-profile donor cows at the Denguin station, including animals from France and Switzerland, such as Duf’Bonus (Duf’ Holstein herd) and Suard Jordan Irene Red (Schrago Frères herd).

In 2015, we collaborated with ECM (France), now part of IMV Imaging, to develop a specialized OPU guide for heifers and cows, in association with Hélène Quinton (Synétics). These innovations significantly improved the precision and safety of ovum pick-up procedures, supporting the expansion of OPU-IVP programs both in our laboratory and directly on farms (Gamarra et al., 2021). Oocyte collections were also conducted in Spain in collaboration with Dr. Daniel Martinez (EMBRIOVET) from Ponderosa Farm, with oocytes transported to our laboratory to complete *in vitro* maturation and subsequent *in vitro* embryo production, followed by cryopreservation (Gamarra et al., 2016). The field application of this technique was also demonstrated in our practices with Lidia cows, resulting in an average yield of four embryos per OPU and a pregnancy rate of approximately 40% in recipient females of the same breed, highlighting the potential of this approach even in breeds exhibiting unique epigenetic characteristics (Gamarra et al., 2017, 2018).

We also investigated the effects of oral propylene glycol (PG) supplementation and anti-Müllerian hormone (AMH) levels on the quantity and quality of follicles, oocytes, and *in vitro* produced embryos through changes in circulating insulin and IGF-1 concentrations, as part of Gamarra-Lacaze’s PhD research, supervised by Andrew Ponter, Claire Ponsart, and Patrice Humblot (Gamarra et al., 2013, 2014, 2015b). In particular, Gamarra et al., demonstrated that short-term PG supplementation improved *in vitro* embryo production in heifers with high AMH concentrations. Further analyses showed that PG administration influenced follicular fluid composition and modulated gene expression in cumulus–oocyte complexes (COCs) and embryos (Gamarra et al., 2017). These studies collectively highlight the utility of AMH as a predictive marker for oocyte yield and embryo developmental potential and the role of metabolic supplementation in optimizing IVP outcomes.

Across multiple dairy and beef breeds, we performed retrospective analyses to evaluate factors affecting OPU-IVP outcomes. Our first study showed that parity significantly influences embryo production in dairy cows, with cows outperforming heifers, whereas no parity effect was observed in beef cows, and that beef cows generally yield more embryos than dairy breeds (Gamarra-Lacaze et al., 2025). Extending these results, a second analysis of 1,176 OPU-IVP sessions examined the impact of repeated OPU, revealing a gradual reduction in recovered oocytes and viable embryos with increasing sessions, while cleavage and blastocyst rates remained stable, indicating that oocyte competence and embryo quality are preserved (Gamarra-Lacaze et al., 2026b). Together, these results highlight how breed, parity, and repeated OPU influence embryo yield and quality, providing essential guidance for optimizing donor management in commercial IVP programs.

By 2026, following several initial on-farm ovum pick-up (OPU) operations, this service is expanding across the Auriva area in southern France, allowing all members to benefit from the latest biotechnologies developed and implemented by Auriva Biotechnology. In particular, our OPU-IVP service enables the rapid multiplication of offspring from elite beef and dairy animals, the use of pregnant cows, and the recovery of genetically valuable cows that do not respond to conventional embryo transfer (ET) treatments. It also allows the use of conventional or sexed semen (female or male), as well as the use of young dairy heifers, based on the objectives of the breeders and the requirements of the selection program.

The foundation of our IVP laboratory techniques since 2009 is based on protocols developed by our national union Eliance, with specific adaptations developed within our laboratory that have enabled us to achieve technically autonomy. Over these years, the integration of OPU, IVP, embryo biopsy (*in vivo* and *in vitro*), the use of sexed semen, and cryopreservation for direct transfer has provided breeders with new opportunities to efficiently multiply and select superior genetic material, both in performance stations and directly on farms.

## Embryo genotyping and biopsy technologies

Even before the advent of genomics, we recognized that the ability to analyze embryos at an early stage would be transformative for cattle breeding. We anticipated that, when genomic technologies became available, the combination of embryo biopsy and cryopreservation would become a cornerstone for genetic selection. Guided by this vision, we began developing and refining these techniques to be ready for practical application.

As early as 1998, we started experimenting with the cryopreservation of biopsied embryos. By 2000, we were offering this service to breeders. Embryo biopsy involves carefully removing a few cells from a developing blastocyst without compromising its viability. These cells can then be analyzed for key genetic traits such as sex, disease status, or performance-related genes.

In 2002, we launched the Aubrac Lait program, focused on multiplying the most productive dairy females. We combined multiple steps: producing embryos, performing biopsies, freezing embryos using the direct transfer method, sexing them, and transferring only female embryos after analysis. This program was carried out in collaboration with Dominic Di Scala and Ludovic Richet (Coopelso), representing a major step in applying advanced reproductive technologies in the field (Lacaze et al., 2016).

The studies of embryo genotyping, initiated by Patrice Humblot through the TYPAGENAE project (2004–2007), was carried out in collaboration with Claire Ponsart and Daniel Le Bourhis (UNCEIA), LABOGENA, INRA, the breeding company UMOTEST, and MIDATEST (Humblot et al., 2010). The project led to a world-first with the birth of Gaston in 2011, the first calf born following the transfer of a genotyped embryo (Figure 3). The integration of embryo biopsy and genomic technologies opened entirely new horizons for genetic selection. In collaboration with UNCEIA, we developed protocols for DNA pre-amplification from embryo biopsy samples, enabling genomic evaluation of embryos with accuracy comparable to that obtained from DNA of live animals (Le Bourhis et al., 2008, 2010; Ponsart et al., 2014). This capability allowed the identification of specific traits at the embryonic stage, including sex, the polled gene, coat color genes, the double-muscling gene, and certain genetic diseases, giving breeders unprecedented control over the next generation.

Since 2012, embryo biopsy has been an essential component of Auriva’s polled breeding programs in the Aubrac and Blonde d’Aquitaine breeds, using the polled gene introgression technique. This approach allows only genetically polled embryos to be transferred, thereby accelerating the fixation of the polled allele (Allais-Bonnet et al., 2013).

**Figure 6:**
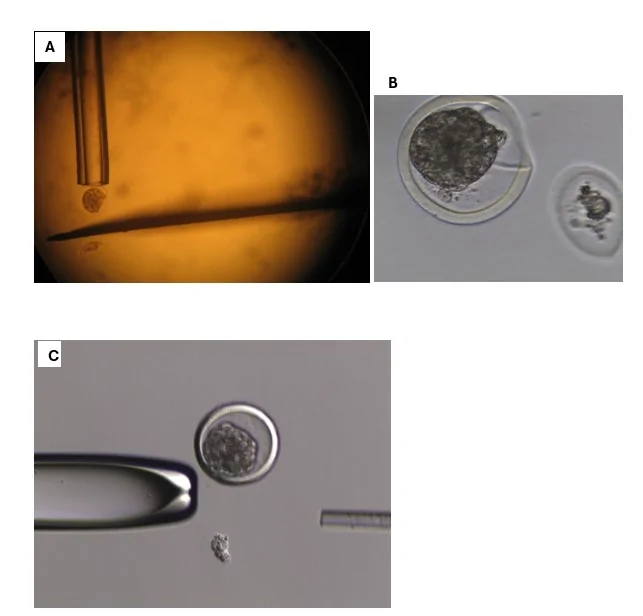
Embryos biopsied using two different techniques: (A and B) Microblade biopsy; (C) Laser-assisted biopsy. Source: Auriva – Elevage (2026).

In 2020, we further refined our embryo biopsy procedure. Since 1991, we had used a microblade technique in which embryos are immobilized by a holding pipette and a steel blade was used to cut 3 to 10 cells (Figures 66B). To reduce embryo damage and improve pregnancy rates, we adopted a laser-assisted biopsy method, similar to techniques used in human IVF laboratories. In this method, a laser precisely opens a small hole in the zona pellucida, allowing 3 to 8 cells to be gently aspirated, thereby minimizing stress to the embryo (Figure 6C). In both laser and microblade techniques, collected cells are stored at -20°C until whole genome amplification (WGA). DNA extraction and WGA were performed using the REPLI-g Single Cell kit (Qiagen©). Biopsied embryos are subsequently frozen using 1.5 M ethylene glycol and 0.1 M sucrose until direct embryo transfer. This innovation led to a significant improvement in pregnancy rates while maintaining optimal call rates, demonstrating both the safety and efficiency of the approach (Gamarra et al., 2023 a, b).

Embryo biopsy can be also used to prevent the appearance of a genetic disease. Axonopathy is a genetic disorder reported in the Blonde d’Aquitaine breed. Although this mutation is not present in Auriva bulls, it persists in the population at a heterozygous frequency of approximately 3% (Blonde Info, 2014). Genetic testing enables determination of the carrier status of animals, as homozygous calves die shortly after birth.

Embryo biopsy combined with genetic analysis provides an effective strategy to prevent the transmission of this defect when genetically valuable heterozygous animals are used in breeding programs, by enabling the identification and selection of non-carrier embryos prior to transfer. This approach was implemented at Auriva Biotech using the cow Niceblonde: 60 embryos were produced by OPU-IVP, biopsied, and genotyped, of which 26 were identified as non-carriers of the mutation. To date, eleven embryos confirmed to be free of the axonopathy mutation have been transferred, resulting in nine pregnancies and three healthy calves born, and the remaining pregnancies are ongoing.

This strategy enables the preservation of valuable genetic lines while effectively avoiding the transmission of the deleterious allele.

## Training our staff: The key to success and the creation of an international exchange network

Over the years, we have organized numerous training sessions to build and maintain expertise in embryo technologies. Techniques related to embryo production are highly specialized and rarely taught in formal training centers, offering few direct career opportunities. Internal training remains the only approach capable of meeting these demanding requirements. The implementation, development, evolution, and adaptation of these techniques to the changing needs of breeders and selection programs depend entirely on the skills and expertise of our team members, regardless of the specific method being applied.

Training for embryo transfer (ET) practitioners and technicians must be comprehensive, encompassing not only technical proficiency but also commercial and interpersonal skills. This ensures high technical performance, supports activity development, and enables effective communication with increasingly knowledgeable breeders, who are often informed through professional networks. Equally important is the continuity of expertise across generations, maintaining and even improving the skill set of the team.

Technicians performing direct embryo transfers are primarily trained inseminators who transfer embryos into recipient cows either in natural estrus or following hormonal synchronization. This procedure is fully integrated into the routine reproductive management of the herd, allowing embryo transfer to be performed efficiently within standard reproductive schedules.

Training of laboratory technicians in IVF techniques is highly demanding and requires exceptional technical skill and precision to ensure consistent and reliable outcomes.

In addition to training our own team, we have welcome numerous masters, engineering, and veterinary students, as well as veterinarians and technicians, offering them the chance to discover embryo biotechnologies and participate in internships, thesis projects, and applied research. These training initiatives are critical not only for the professional development of participants but also for advancing Auriva’s programs, promoting knowledge dissemination, collaboration, and the integration of innovative approaches into routine practice, strengthening both our team and the broader reproductive biotechnology community.

## International technical support and field applications

Beyond research and breeding programs in France, our team has provided technical support for international embryo transfer projects in Chile, Mexico, Peru, Brazil, Colombia, Vietnam, China, Hungary, Spain, Cameroon, Thailand, New Caledonia, and Réunion Island.

A notable example is the genetic program implemented on Réunion Island, where more than 1,000 beef embryos derived from French mainland breeds (Limousin, Blonde d’Aquitaine, Charolais, Aubrac, and Salers) were transferred under extensive tropical conditions, achieving pregnancy rates exceeding 55% (Gamarra-Lacaze et al., 2026a forthcoming).

Furthermore, our team collaborates with Auriva’s commercial division, Synetics World, providing technical and genetic expertise to support the development of new international markets for semen and embryo exports.

## Conclusion: Anticipating needs and preparing the future today

Over the past forty years, reproductive biotechnologies have evolved from experimental procedures into indispensable tools for accelerating genetic progress in cattle breeding. Embryo transfer, OPU-IVP, genomic selection, and embryo genotyping now enable the improvement of both quantitative traits (e.g., growth and milk yield) and qualitative traits (e.g., health, conformation, functional traits, and robustness), while maintaining high sanitary and genetic standards and supporting more sustainable cattle breeding, and I believe that genetic selection based on genomic testing of embryo biopsies will play a central role.

The ultimate success of these technologies depends not only on technical innovation but also on the people who implement them: practitioners, laboratory technicians, managers, researchers, and breeders. Strong national cooperative structures, continuous training of specialized personnel, and active scientific exchanges have been essential to their development. Participation in international congresses and workshops particularly within professional communities such as AETE has provided invaluable opportunities to share experiences, discuss emerging techniques, and build lasting scientific networks. Indeed, attending AETE meetings has allowed me to remain closely engaged with colleagues, exchange knowledge, and stay at the forefront of reproductive science.

Looking toward the future, the challenge is to anticipate the evolving needs of breeding programs and continue adapting reproductive biotechnologies accordingly. By fostering strong scientific networks, promoting active engagement in international forums, and supporting the training and development of new generations, we can ensure that these technologies remain effective, relevant, and capable of contributing to sustainable genetic progress in cattle production.

Being recognized today by AETE is deeply meaningful. This award reflects not only my professional experience but also the dedication, expertise, and collaboration of countless practitioners, colleagues, mentors, and students I have had the privilege to work with over the years. It is a reminder that the progress we celebrate is the result of a shared passion for innovation and a collective commitment to advancing reproductive science.

## Data Availability

Research data is available in the body of the article.
